# Simvastatin attenuates silica-induced pulmonary inflammation and fibrosis in rats via the AMPK-NOX pathway

**DOI:** 10.1186/s12890-024-03014-9

**Published:** 2024-05-08

**Authors:** Cunxiang Bo, Fang Liu, Zewen Zhang, Zhongjun Du, Haidi Xiu, Zhenling Zhang, Ming Li, Caiqing Zhang, Qiang Jia

**Affiliations:** 1 First Clinical Medical College, Shandong University of Traditional Chinese Medicine, Jinan, Shandong China; 2 Shandong Academy of Occupational Health and Occupational Medicine, Shandong First Medical University & Shandong Academy of Medical Sciences, Jinan, Shandong China; 3 Guangzhou Huaxia Vocational College, Guangzhou, China; 4 Department of Radiology, Shandong Provincial Hospital Affiliated to Shandong First Medical University, Jinan, Shandong China; 5 Pulmonary and Critical Care Medicine, Shandong Province’s Second General Hospital (Shandong Province ENT Hospital), Shandong University of Traditional Chinese Medicine, Jinan, Shandong Province, Shandong China

**Keywords:** Simvastatin, Pulmonary fibrosis, Oxidative stress, Epithelial mesenchymal transformation, AMPK, NOX

## Abstract

**Background:**

Simvastatin (Sim), a hydroxy-methylglutaryl coenzyme A (HMG-CoA) reductase inhibitor, has been widely used in prevention and treatment of cardiovascular diseases. Studies have suggested that Sim exerts anti-fibrotic effects by interfering fibroblast proliferation and collagen synthesis. This study was to determine whether Sim could alleviate silica-induced pulmonary fibrosis and explore the underlying mechanisms.

**Methods:**

The rat model of silicosis was established by the tracheal perfusion method and treated with Sim (5 or 10 mg/kg), AICAR (an AMPK agonist), and apocynin (a NOX inhibitor) for 28 days. Lung tissues were collected for further analyses including pathological histology, inflammatory response, oxidative stress, epithelial mesenchymal transformation (EMT), and the AMPK-NOX pathway.

**Results:**

Sim significantly reduced silica-induced pulmonary inflammation and fibrosis at 28 days after administration. Sim could reduce the levels of interleukin (IL)-1β, IL-6, tumor necrosis factor-α and transforming growth factor-β1 in lung tissues. The expressions of hydroxyproline, α-SMA and vimentin were down-regulated, while E-cad was increased in Sim-treated rats. In addition, NOX4, p22pox, p40phox, p-p47phox/p47phox expressions and ROS levels were all increased, whereas p-AMPK/AMPK was decreased in silica-induced rats. Sim or AICAR treatment could notably reverse the decrease of AMPK activity and increase of NOX activity induced by silica. Apocynin treatment exhibited similar protective effects to Sim, including down-regulating of oxidative stress and inhibition of the EMT process and inflammatory reactions.

**Conclusions:**

Sim attenuates silica-induced pulmonary inflammation and fibrosis by downregulating EMT and oxidative stress through the AMPK-NOX pathway.

**Supplementary Information:**

The online version contains supplementary material available at 10.1186/s12890-024-03014-9.

## Background

Silicosis is one of the most serious occupational diseases in the world due to irreversible pulmonary fibrosis [1], and its pathogenesis is very complex and not been fully elucidated at present. Its main pathological features include persistent pulmonary inflammation and progressive pulmonary fibrosis. The occurrence of silicosis originates from inhalation of free silica into alveolar macrophages, consequently producing pro-inflammatory and pro-fibrotic factors to trigger inflammation and irreversible fibrosis [2]. In addition, persistent inflammation, oxidative stress (OS), epithelial mesenchymal transformation (EMT) and extracellular matrix (ECM) deposition also contribute to the development of silicosis [2]. EMT is pathologically characterized as the transformation of epithelial cells into mesenchymal cells, activating the transcription factors and increasing the ECM accumulation. Multiple evidence has indicated that OS induced by excessive reactive oxygen species (ROS) plays an important role in the progression of silicosis [3–4]. Moreover, ROS accumulation has been reported to involve in the progression of lung fibrosis by inducing epithelial cell damage, promoting the release of cytokines, and accelerating the EMT process [5–6]. Nicotinamide adenine dinucleotide phosphate (NADPH) oxidase (NOX), a major source of ROS in many organs, is involved in many key physiological processes, including host defense, post-translational modification of proteins, cell signaling and regulation of gene expression. So far, seven isoforms of NOX catalytic components have been identified, including NOX1-5 and durox 1–2 [7], among which NOX2 and NOX4 are mainly expressed in the lungs. The NOX2 protein complex is composed of two membrane proteins (NOX2 and p22phox) forming the catalytic core, three cytosolic proteins (p67phox, p47phox and p40phox) and a small GTPase Rac. NOX2 activation depends on the phosphorylation of cytosolic subunits. The p47phox regulatory subunit plays a key role in acute activation of NADPH oxidase [8]. After phosphorylation, p47phox interacts with p40phox and p67phox subunits and migrates to a plasmatic membrane to form the enzymatic complex [9–10]. NOX4 is composed of membrane subunits NOX4 and p22phox, but its activation has not been illustrated clearly. It has been reported that NOX4 expression is increased in pulmonary fibroblasts, and siNOX4 can inhibit bleomycin-induced pulmonary fibrosis in mice [11]. Moreover, myofibroblast activation and ECM accumulation can be inhibited by down-regulating NOX2 and NOX4 levels in alveolar macrophages of radiation-induced fibrosis mice [12]. The role of NOX in acute respiratory distress syndrome and chronic obstructive pulmonary disease has been widely studied [13], but is still unclear in silica-induced pulmonary fibrosis.

Adenosine 5’-monophosphate (AMP)-activated protein kinase (AMPK), a member of the Ser/Thr kinase family expressed in various organs, is a key signaling molecule in the regulation of bioenergy metabolism. AMPK could regulate autophagy, cellular stress resistance, inflammatory response and EMT processes through regulation of multiple downstream targets, such as mTOR, NF-κB and TGF-β1 signaling [14–15]. Lower AMPK activity is a risk factor for the development of organ fibrosis such as the heart, kidney, and lungs [16–18]. It has been reported that the proliferation and migration of myocardial fibroblasts are enhanced in AMPKα1 knockout mice [19]. AMPKα2 deficiency enhances EMT and inflammatory infiltration in a mouse model of unilateral ureteral obstruction [20], while its activation inhibits EMT processes to reduce renal fibrosis [21]. Additionally, AMPK activity is also reduced in humans with idiopathic pulmonary fibrosis (IPF) and experimental mouse models of lung fibrosis [22]. An AMPK activator metformin exerted protective effects on the development of fibrosis by decreasing TGF-β1-induced α-SMA, fibronectin expression, collagen type I expression, and myofibroblast differentiation [23–25]. Substantial studies confirmed that AMPK activation attenuated pulmonary inflammation and fibrosis by inhibiting TGF-β1 signaling and activating autophagy through the AMPK-mTOR pathway [25–26]. Interestingly, a recent study has shown that AMPK activation negatively regulates NOX expression to reduce neointimal hyperplasia in diabetic rats [27]. Therefore, we hypothesized that silica exposure contributed to the development of silica-induced pulmonary fibrosis by promoting the EMT process partly through AMPK-NOX signaling.

Simvastatin (Sim), a hydroxy-methylglutaryl coenzyme A (HMG-CoA) reductase inhibitor, is widely used in the prevention and treatment of cardiovascular diseases, and has a variety of biological activities, such as anti-inflammation and anti-fibrosis [28–29]. Sim can not only prevent cardiac fibrosis following myocardial infarction, alleviate ultrastructural damage and improve heart function [30], but also attenuate bleomycin-induced pulmonary inflammation and fibrosis in mice via down-regulation of TNF-α and TGF-β1 expression, suggesting its potential in anti-fibrosis [31]. Furthermore, Sim exerts various effects via AMPK activation [32–33]. Based on the anti-fibrotic potential of Sim, we gambled Sim may protect against pulmonary fibrosis by regulating AMPK-NOX signaling in silicosis. In this study, we established the rat models of silicosis by intratracheal instillation and treatment with Sim. The results demonstrated that Sim could alleviate silica-induced pulmonary inflammation and fibrosis, which may be potentially attributed to activation of AMPK and down-regulation of NOX-derived ROS production.

## Materials and methods

### Reagents

Silica powder (SiO_2_, 80% 1–5 μm, S5631, Sigma-Aldrich, USA) was added distilled water for preparing 50 mg/mL silica suspension. Sim (MSD, UK) and AICAR (AMPK agonist, Abmole, USA) were dissolved with DMSO and added distilled water for preparing 1 mg/kg AICAR solution. Apocynin (NOX inhibitor, purity > 99%, Aoodq, USA) was dissolved with DMSO (Sigma-Aldrich, USA) and added distilled water for preparing 50 mg/kg Apocynin solution. Other reagents included hydroxyproline (HYP), superoxide dismutase (SOD) and ROS kits (Nanjing Jiancheng, Nanjing, China); malondialdehyde (MDA) and glutathione peroxidase (GSH-Px) detection kits (Shanghai Biotechnology Co., LTD.); tumor necrosis factor (TNF)-α, interleukin (IL)-1β and IL-6 enzyme-linked immunosorbent assay (ELISA) kits (Beijing Baizhi Biological Technology Co., LTD.); transforming growth factor (TGF)-β1, E-cad, Vimentin, α-SMA and NOX2 antibodies (Abcam, London, England, 1:1 000); NOX4, p47phox and p40phox antibodies (Proteintech, China, 1:1 000); p-p47phox antibodies (Bioworld, USA, 1:1 000); p22phox antibodies (CST, USA, 1:1 000); GAPDH antibodies (Shanghai Beyotime Biotechnology, China, 1:1 000); IRDye680 Sheep Anti-Rabbit secondary antibodies and aRD Sheep Anti-Mouse secondary antibodies (LI-COR, USA, 1:10 000).

### Animals and treatment

Thirty-six healthy male Sprague-Dawley rats, weighing 180–200 g and aged 8–10 weeks, were purchased from Jinan Pengyue Experimental Animal Breeding Co. Ltd (Jinan, China). They were allowed free access to food and water in a specific pathogen-free (SPF)-grade animal facility with temperature of 20–26℃ and humidity of 40-70%, and were randomly divided into 6 groups with 6 in each, including: control, silica, AICAR (AMPK agonist), apocynin (NOX inhibitor) and Sim (5 or 10 mg/kg) groups. The rats in the control group were given 1.0 mL normal saline, while those in other groups were given 1.0 mL silica suspension (50 mg/mL) with a single intratracheal instillation as previously described [34]. After 24 h, AICAR group was given intraperitoneal injection of AICAR solution (1 mg/kg/day) and apocynin group received 50 mg/kg apocynin solution 3 times per week [35–37]. The Sim group received orally administration of 5 or 10 mg/kg Sim solution once a day [38]. The control group and silica group received the equal volume of 0.9% sodium chloride once a day. After 28 days, 6 rats in each group were anesthetized by 3% pentobarbital sodium and sacrificed, and the blood and lungs of each rat were collected for further studies. This animal study was approved by the Medical Ethics Committee of Shandong Academy of Occupational Health and Occupational Medicine (Protocol Number: SDZFY-EC-A2022-03).

### Blood lipid assays

Sim is common clinical cholesterol-lowering drugs. To assess the safety of Sim on silica-induced rats, the levels of total cholesterol (TCHO), triglycerides (TG), low density lipoprotein cholesterol (LDL-C) and high-density lipoprotein cholesterol (HDL-C) were detected using Automatic Biochemical Analyzer (TBA-120FR, Japan). To obtain the serum, the whole blood was collected without anticoagulants and left at 37 °C for 30 min. Serum was separated by centrifugation for 15 min at 3 500 rpm. 

**Lung histological examinations**.

Lung coefficients were used to evaluate the damage of lungs in each group. The calculation formula was as follows: Lung coefficient = wet lung weight (g)/body weight (g) *100%. Right upper lung tissues were fixed with 4% paraformaldehyde solution, dehydrated, and embedded in paraffin, and then cut into 4 µM slices for hematoxylin and eosin (H&E) and Masson staining to assess the degree of inflammatory infiltration and pulmonary fibrosis by Szapiel’s method [39] under the light microscope (Nikon Corporation, Japan).

### HYP assays

100 mg lung tissues from each rat were measured according to the instructions of HYP assay kits. The absorbance value was measured at a wavelength of 550 nm, and the HYP content in the lung tissue was calculated as µg of HYP per mg of protein.

### ELISA assays

100 mg lung tissues from each rat were homogenized and frozen at -20℃ overnight. They were taken out and repeatedly frozen, then thawed twice to break the cell membrane in the second day. After centrifugation at 5 000 g at 4℃ for 5 min, the supernatant was extracted. The expression levels of IL-1β, IL-6 and TNF-α in lung tissues were detected according to ELISA kit instructions.

### OS indicators

100 mg lung tissues from each rat were homogenized in phosphate buffer saline on ice, then centrifugated at 10 000–12 000 g for 10 min. The supernatant was taken. Activities of SOD, GSH-Px and MDA in lung tissues were measured according to the instructions of the detection kits.

### Detection for NOX and ROS activity

The activity of NOX was determined by chemiluminescence methods [40]. Briefly, the lung tissue was weighed, homogenized, centrifuged at 12 000 g for 20 min at 4 ℃, and then supernatant was extracted. Protein concentration was determined by BCA assay kits. 5 μm lucigenin and 100 µM NADPH were added to the supernatant and incubated at 37℃ for 10 min in the dark, the values were read by FLUOstar Omega multi-functional microplate reader. NOX activity was expressed as relative light units per minute per milligram protein. According to the ROS assay kit, 100 mg of the rat lung tissue was taken, and single-cell suspension was prepared by enzymatic digestion methods. ROS was labeled with a fluorescent probe DCFH-DA, and the value was read by FLUOstar Omega multi-functional microplate reader. ROS activity was measured as relative light units/mg protein.

### Western blotting analysis

Lung tissues were weighed and homogenized, centrifuged at 12 000 r/min for 20 min at 4℃, and the supernatant was obtained. The protein concentration in the supernatant was determined by the BCA method. 30 ug samples per well were separated on sodium dodecyl sulfate polyacrylamide gel and transferred to polyvinylidene fluoride membrane. After blocking with 5% skim milk for 1 h, the membrane was washed 4 times, 5 min each time. The blots were cut prior to hybridization with antibodies during blotting. The antibodies of TGF-β1, α-SMA, Vimentin, E-cad, NOX2, NOX4, p22pox, p40phox, p47phox, p-p47phox, AMPK, p-AMPK and GAPDH were added, overnight at 4℃. The membrane was washed for 4 times, then corresponding fluorescent secondary antibodies were added and incubated for 1 h. Western blotting was analyzed with Odyssey®CLx two-color infrared laser imaging system and Image Studio software.

### Statistical analysis

All data were expressed as the mean ± standard deviation and analyzed by GraphPad Prism 8.0 statistical software. The Bartlett’s test was used to test the homogeneity of variance. One-way analysis of variance was used for comparison between groups and the Dunnett’s test was used for comparison between two groups. *p* < 0.05 was considered statistically significance.

## Results

### Effect of Sim on blood lipid levels in silica-induced rats

**Fig. 1:**
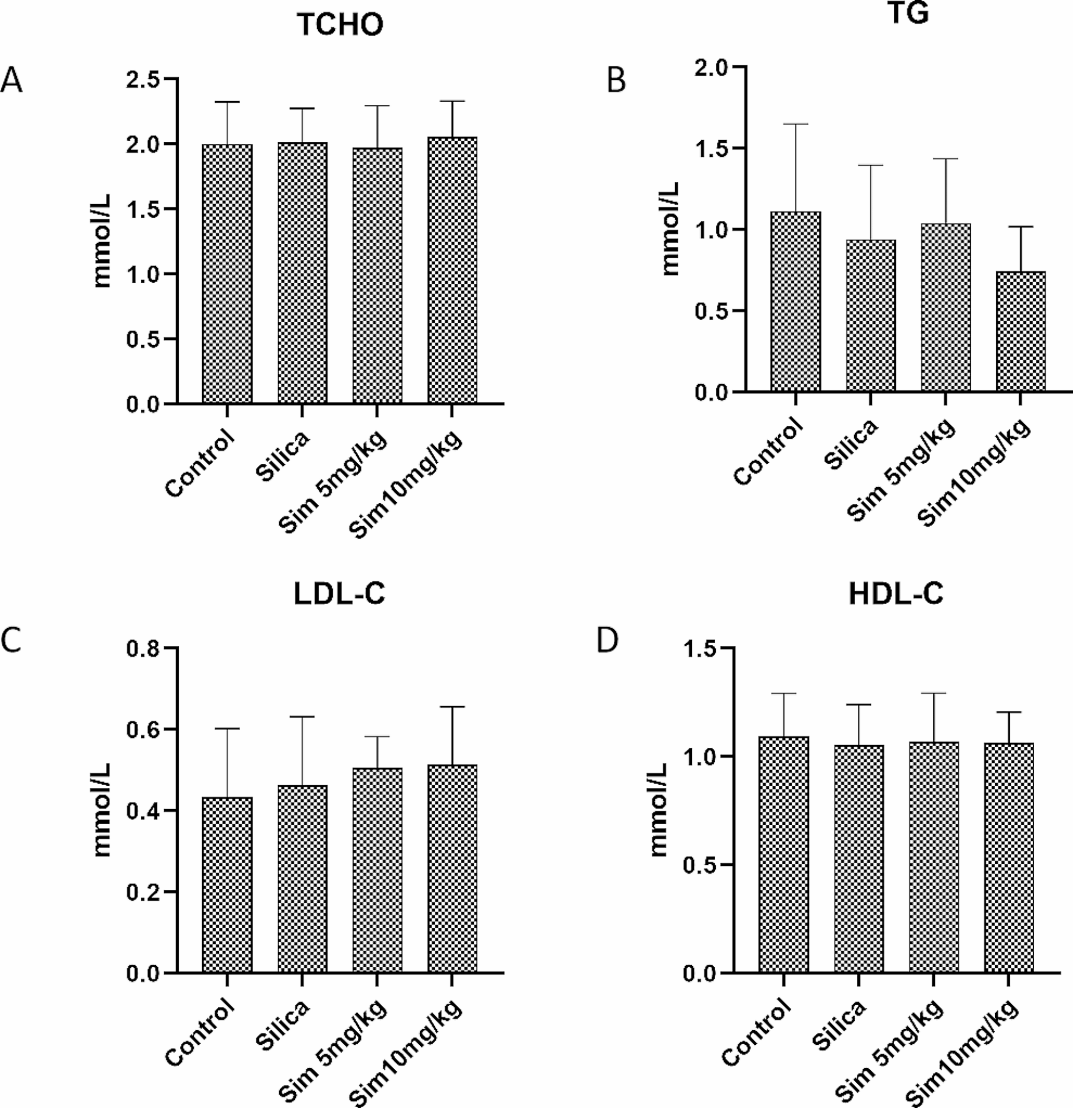
Effects of Sim treatment on blood lipid levels in silica-induced rats. The levels of TCHO (**A**), TG (**B**), LDL-C (**C**), and HDL-C (**D**). Data are presented as the means ± SD, *n* = 6

To evaluate the effect of Sim on blood lipid levels in silica-induced rats, the serum levels of TCHO, TG, LDL-C and HDL-C were detected. These results showed that there were no significant differences among the control, silica, and Sim groups (Fig. 1), indicating that Sim had no negative effects on the blood lipid of silicosis rats, with good safety.

### Alleviation of lung injury and inflammation through Sim treatment

**Fig. 2:**
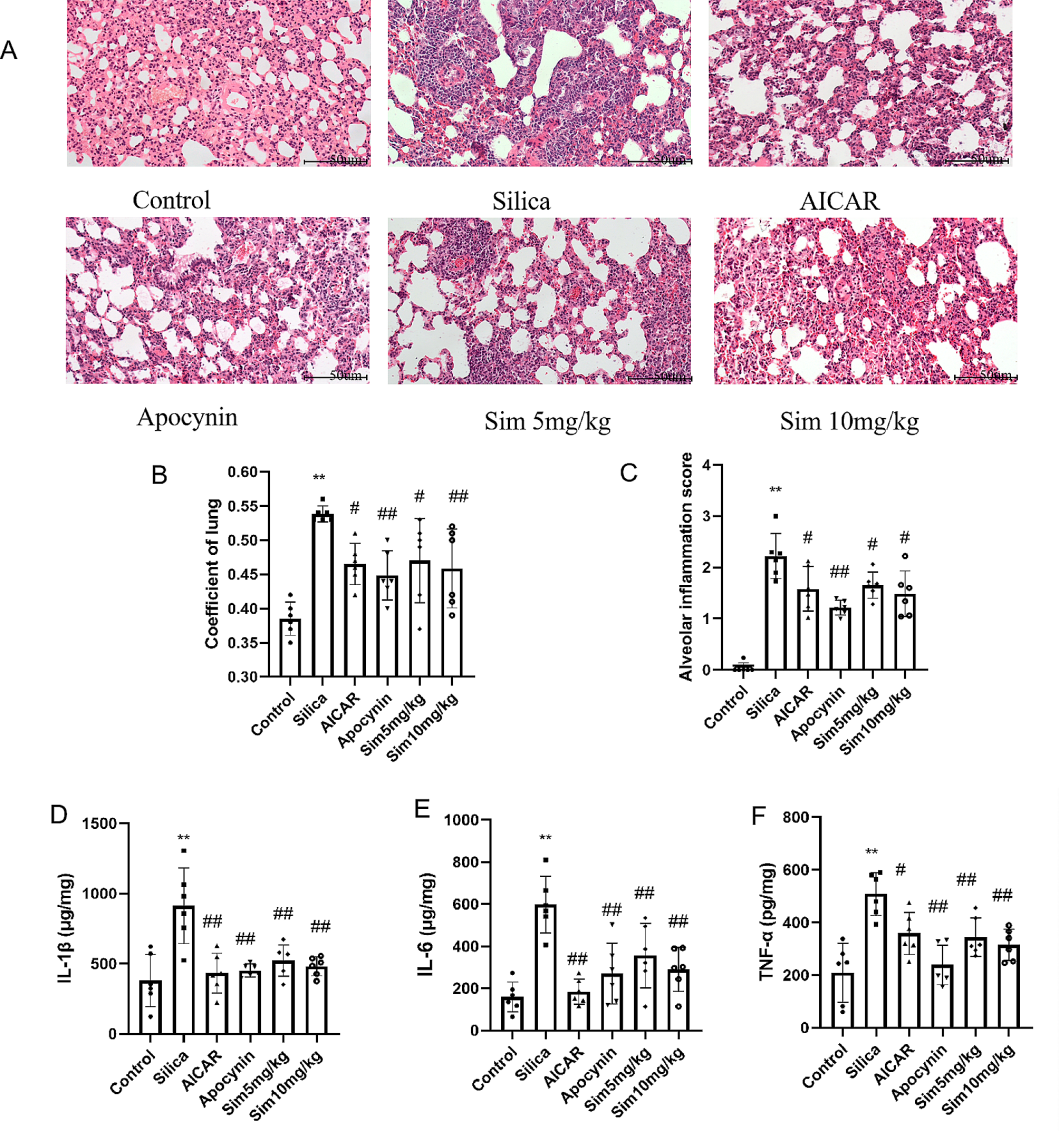
Effects of Sim treatment on the lung coefficients and inflammation in silica-induced rats. (**A**) H&E staining (200×, *n* = 6). (**B**) The lung coefficients. (**C**) Alveolar inflammation scores quantified by H&E staining. The levels of IL-1β (**D**), IL-6 (**E**) and TNF-α (**F**) in lung tissues. Data are presented as the means ± SD, *n* = 6. *p < 0.05, **p < 0.01 vs. the control group; ^#^*p* < 0.05, ^##^*p* < 0.01 vs. the silica group

To investigate the protective effect of Sim on lung tissues induced by silica, the lung coefficient was measured. The results showed that the lung coefficient was increased significantly in the silica group compared with the control group, while the treatment with Sim (5, 10 mg/kg) significantly decreased the lung coefficient compared with the silica group (*p* < 0.05; Fig. 2B). H&E staining was used to assess the therapeutic effect of Sim treatment on silica-induced lung damage. It could be observed normal lung tissue structures and intact alveolar structures in the control group, while obvious pathological damages in lung tissues of the silica group, accompanying by the thickened alveolar septum and significant inflammatory cell infiltration. However, treatment with Sim (5, 10 mg/kg) could remarkably reduce the thickened alveolar septum and obvious inflammatory cell infiltration compared with silica group (*p* < 0.01; Fig. 2A and C). Through ELISA, it was further found that the levels of pro-inflammatory cytokines, such as IL-1β, TNF-α and IL-6 were obviously increased in the silica group compared with the control group. Meanwhile, treatment with Sim (5, 10 mg/kg) meaningfully reduced the levels of IL-1β, TNF-α and IL-6 in lung tissues after exposure to silica for 28 days (*p* < 0.05 or *p* < 0.01; Fig. 2D-F). All these results revealed that Sim treatment could alleviate the lung damage and inflammation in silica-induced rats. This protective effect also appeared in AICAR or apocynin group.

### Attenuation of lung fibrosis through Sim treatment

**Fig. 3:**
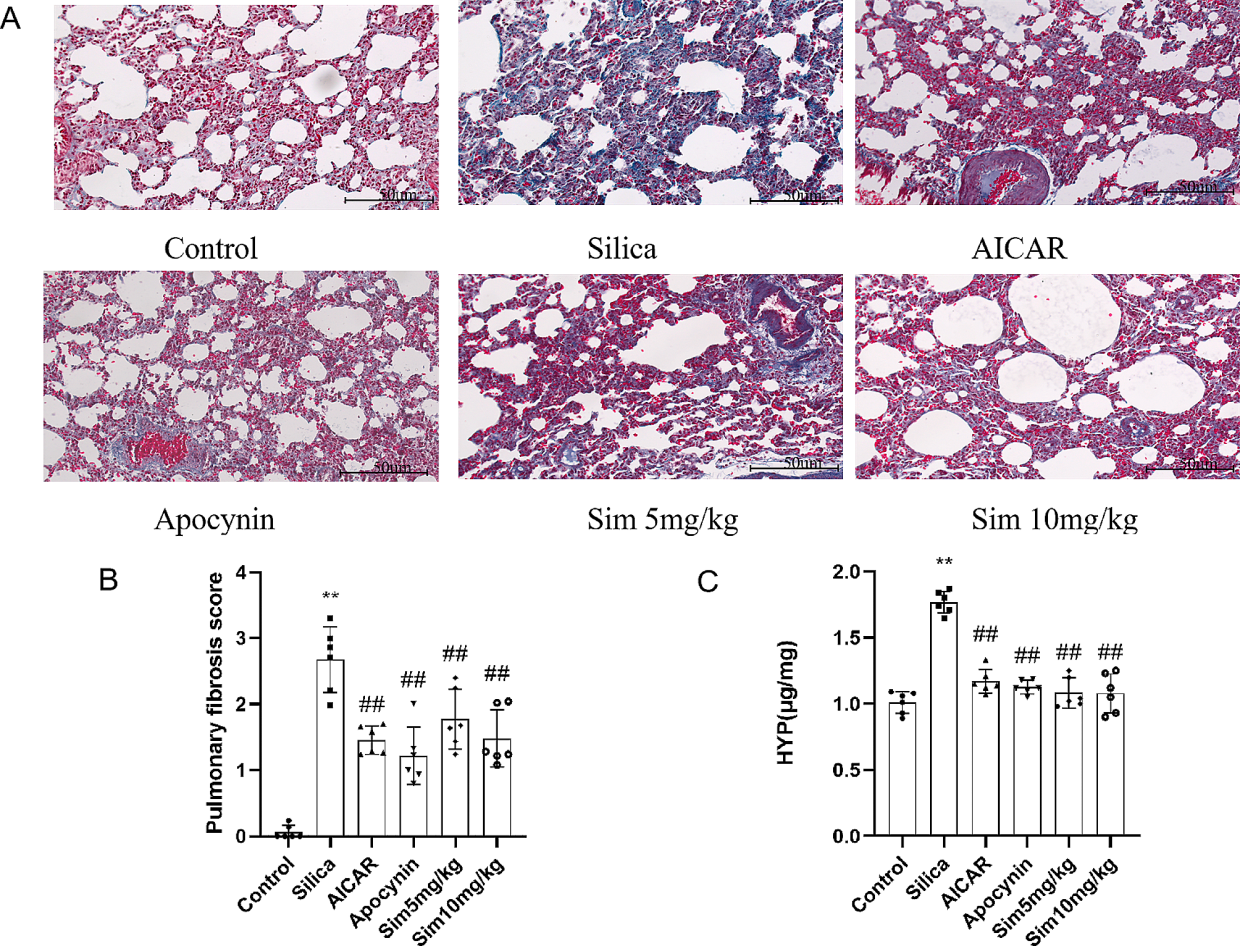
Effects of Sim treatment on the lung fibrosis in silica-induced rats. (**A**) Masson staining (200×). (**B**) Pulmonary fibrosis quantified by Masson staining. (**C**) The level of HYP in lung tissues. Data are presented as the means ± SD, *n* = 6. **p < 0.01 vs. the control group; # *p* < 0.05, ##*p* < 0.01 vs. the silica group

Progressive pulmonary fibrosis is the main pathological feature in the advanced stage of silicosis. To confirm whether Sim attenuated silica-induced lung fibrosis in rats, Masson staining was used to assess the degree of lung fibrosis. It could be observed that there were normal lung tissue structures and few blue collagens in the control group, while thickened alveolar walls and obvious blue collagen deposition in silica group (Fig. 3A). Meanwhile, Masson staining also showed that the pulmonary fibrosis score of the silica group was significantly higher than that of the control group. Surprisingly, after treatment with Sim (5, 10 mg/kg) and AICAR or apocynin for 28 days, thickened alveoli, blue collagens deposition and pulmonary fibrosis scores were all notably decreased compared with the silica group (*p* < 0.05 or *p* < 0.01; Fig. 3A and B). To further study the anti-fibrosis effect of Sim on silica-induced rats, HYP, a special amino acid in collagen, was observed in lung tissues. As shown in Fig. 3C, there was over-expression of HYP in the silica group compared with the control group, and treatment with Sim (5, 10 mg/kg), AICAR or apocynin remarkably decreased the levels of HYP in lung tissues of the rats. Collectively, Sim treatment could attenuate silica-induced lung fibrosis in rats.

### Inhibition of the EMT process in lung tissues by Sim treatment

**Fig. 4:**
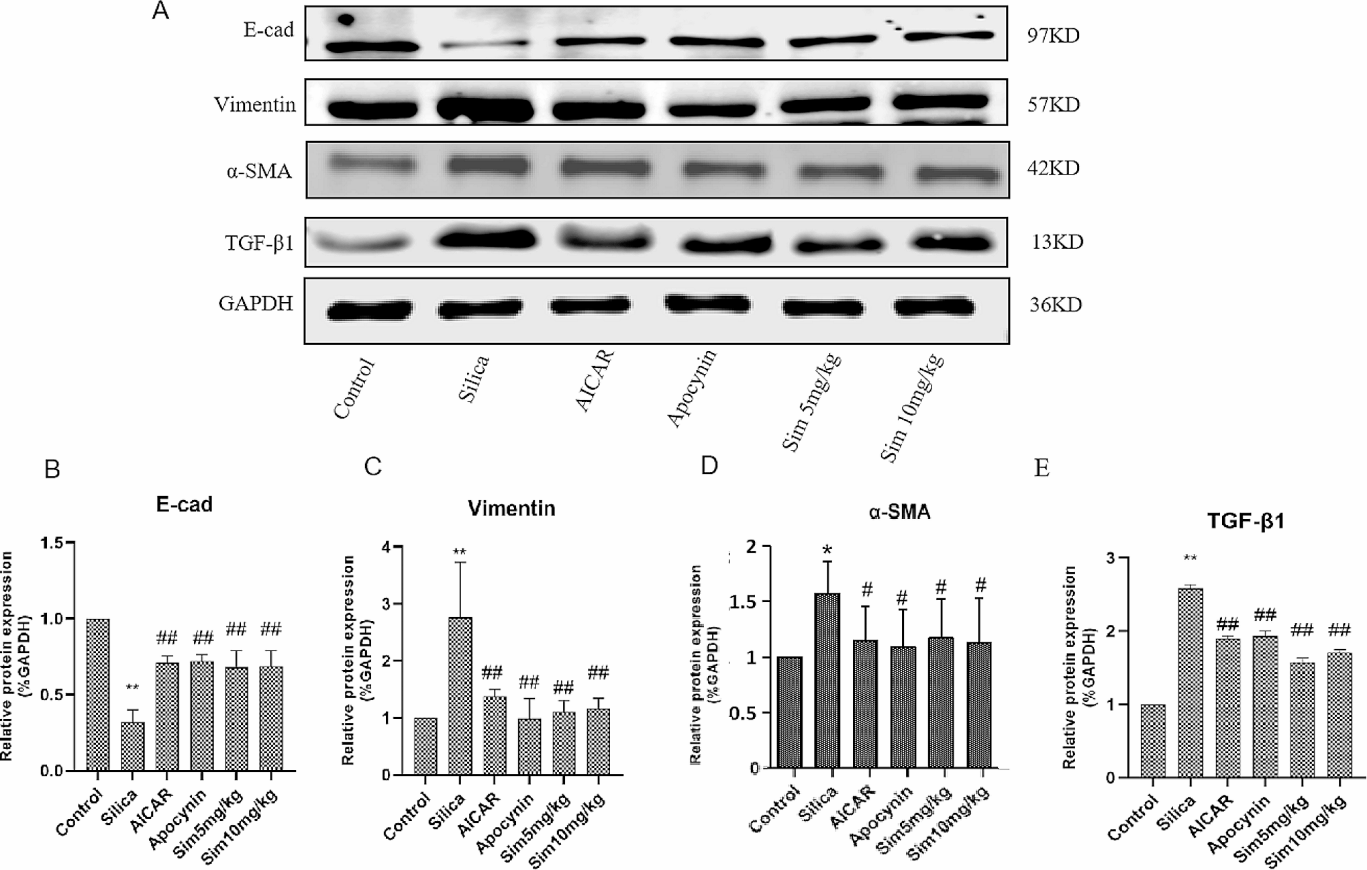
Effects of Sim treatment on the EMT in silica-induced rats. (**A**) The western blotting bands of E-cad, Vimentin, α-SMA, TGF-β1 and GAPDH in lung tissues among different groups (*n* = 3). The samples were derived from the same experiment, and the gels/blots were processed in parallel. The gels/blots are corpped, and full-length gels/blots are presented in Fig. S1. The relative expressions of E-cad (**B**), Vimentin (**C**), α-SMA (**D**), and TGF-β1 (**E**). Data are presented as the means ± SD, *n* = 3. **p < 0.01 vs. the control group; ^#^*p* < 0.05, ^##^*p* < 0.01 vs. the silica group

Activation of the EMT process may be the key mechanism of lung fibrosis. To investigate the mechanism of Sim against fibrosis, we examined the EMT biomarkers including E-cad, α-SMA and Vimentin by western blotting assays. Our studies showed that the expression of E-cad was highly decreased while α-SMA and Vimentin expressions were increased in the silica group compared with the control group. Meanwhile, Sim treatment (5, 10 mg/kg) significantly up-regulated the expression of E-cad, while down-regulated the expressions of α-SMA and Vimentin (*p* < 0.05 or *p* < 0.01; Fig. 4A-D and Fig. S1). Moreover, we also explored the expression of TGF-β1, a major cytokine inducing EMT in lung tissues. As expected, the expression of TGF-β1 was over-expressed in silica-induced rats, and Sim treatment (5, 10 mg/kg) significantly decreased the level of TGF-β1 in lung tissues of silica-induced rats (*p* < 0.05 or *p* < 0.01; Fig. 4A, E and Fig. S1). AICAR or apocynin treatment also up-regulated E-cad expression, but down-regulated α-SMA, Vimentin and TGF-β1 expressions in silica-induced lung tissues. All these findings indicated that Sim alleviated pulmonary fibrosis by inhibiting TGF-β1-induced EMT process in silica-induced rats.

### Alleviation of OS in lung tissues via Sim treatment

**Fig. 5:**
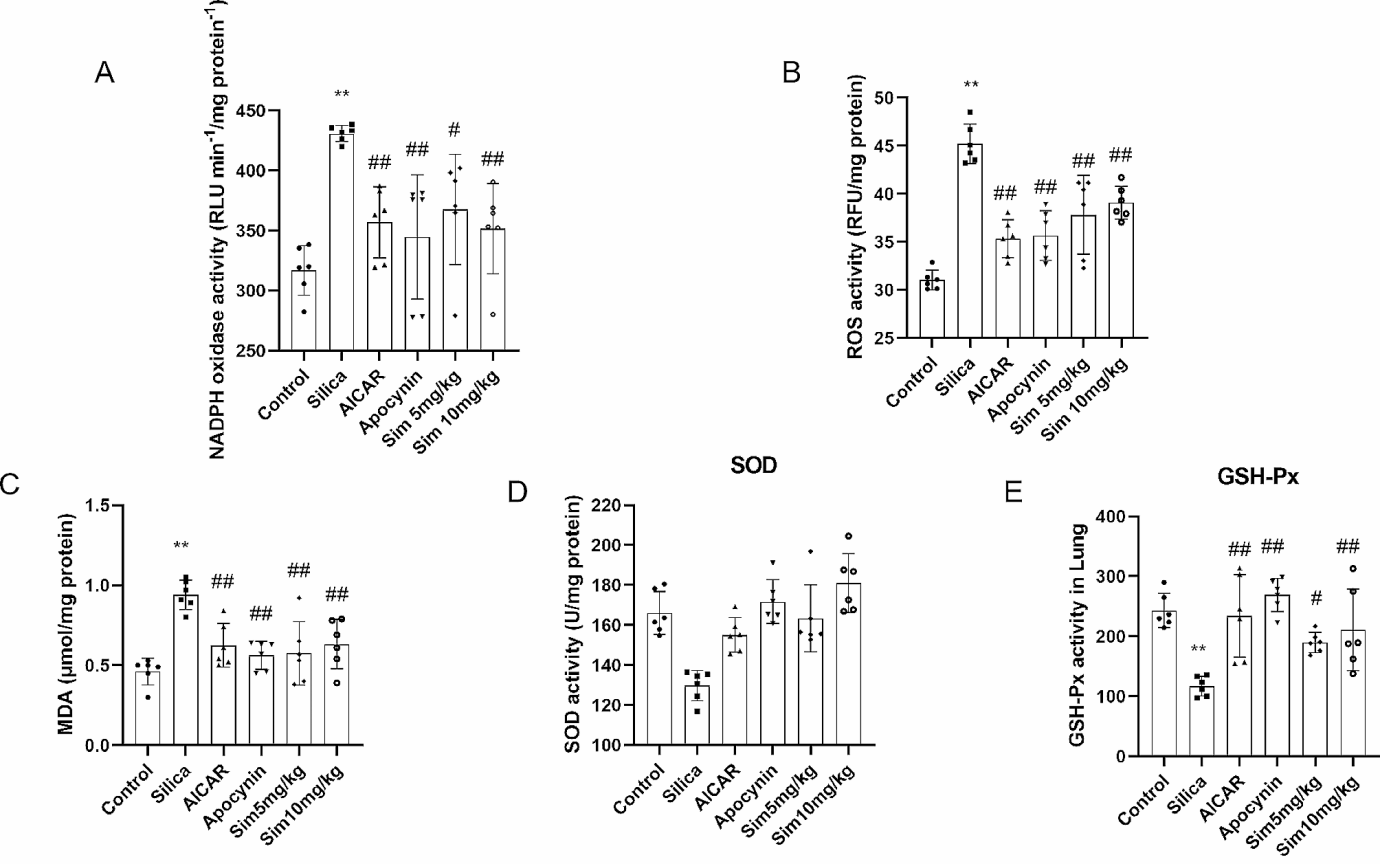
Effects of Sim on OS in lung tissues of silica-induced rats. The activities of NOX (**A**), ROS (**B**), MDA (**C**), SOD (**D**), and GSH-Px (**E**) in lung tissues. Data are presented as the means ± SD, *n* = 6. *p < 0.05, **p < 0.01 vs. the control group; ^#^*p* < 0.05, ^##^*p* < 0.01 vs. the silica group

OS is a vital factor to promote the development of silicosis. To determine the effect of Sim on OS in silica-induced rats, we observed a battery of OS indicators and their regulations. Compared with the control group, the level of MDA (a product of OS) [41] in lung tissues was significantly increased in the silica group. Interestingly, after Sim treatment, the level of MDA was remarkably reduced in lung tissues (*p* < 0.05 or *p* < 0.01; Fig. 5C). SOD and GSH-Px as classical antioxidant enzymes were assessed as anti-OS markers [42]. Compared with the control group, the activities of SOD and GSH-Px were significantly reduced in lung tissues after exposure to silica for 28 days. Meanwhile, Sim treatment (5, 10 mg/kg) remarkably up-regulated the activities of SOD and GSH-Px in lung tissues compared with the silica group (*p* < 0.05 or *p* < 0.01; Fig. 5D and E). Cumulatively, these results demonstrated that Sim may have anti-OS effects in lung tissues of silica-induced rats.

To confirm the anti-OS effect of Sim, ROS activity was measured by a fluorescent probe DCFH-DA in lung tissues. The results showed that ROS activity was highly increased after silica exposure, but significantly decreased after treatment with Sim (5, 10 mg/kg) (*p* < 0.05 or *p* < 0.01; Fig. 5B). Additionally, to further explore the activity of NOX, we added apocynin (a NOX inhibitor) group and found that NOX activity was increased significantly after exposure to silica for 28 days, while decreased remarkably after Sim treatment. Meanwhile, the treatment with apocynin had similar effects to Sim (*p* < 0.05 or *p* < 0.01; Fig. 5A). These results indicated that Sim may exert therapeutic effects by down-regulating NOX-derived ROS production.

### Regulation of AMPK-NOX pathway in lung tissues by Sim treatment

**Fig. 6:**
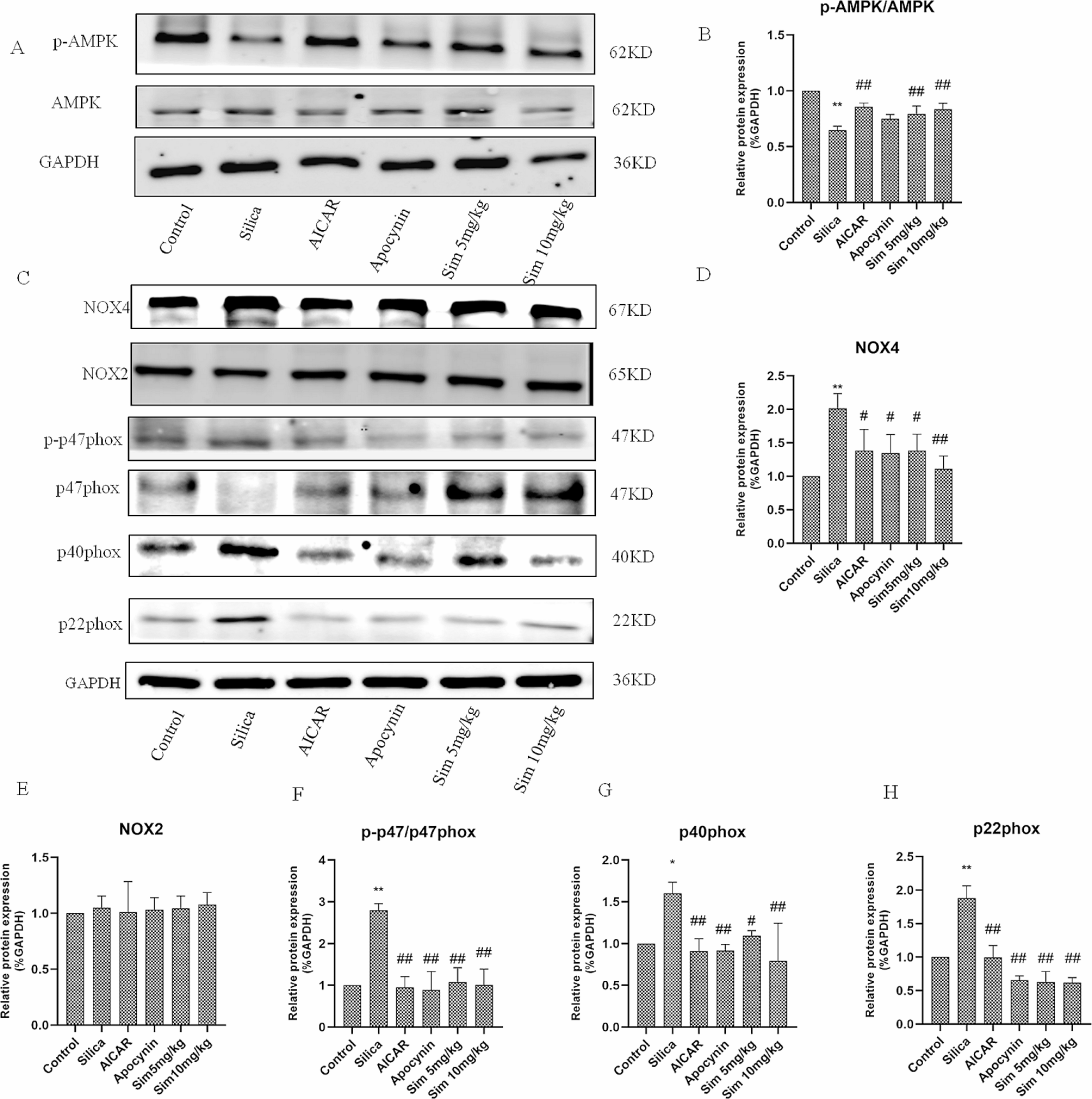
Effects of Sim treatment on the AMPK-NOX pathway in silica-induced rats. (**A**) The western blotting bands of p-AMPK, AMPK and GAPDH in lung tissues among different groups (*n* = 3). The gels/blots are corpped, and full-length gels/blots are presented in Fig. S2. (**B**) The relative protein expression of p-AMPK/AMPK. (**C**) The western blotting bands of NOX4, NOX2, p-p47phox, p47phox, p40phox, p22phox and GAPDH in lung tissues among different groups (*n* = 3). The gels/blots are corpped, and full-length gels/blots are presented in Fig. S3. The relative protein expressions of NOX4 (**D**), NOX2 (**E**), p-p47phox/p47phox (**F**), p40phox (**G**), and p22phox (**H**). Data are presented as the means ± SD, *n* = 3. *p < 0.05, **p < 0.01 vs. the control group; ^#^*p* < 0.05, ^##^*p* < 0.01 vs. the silica group

Sim plays a pharmacological role by activating AMPK expression, which has been reported to be a possible treatment for fibrosis. To further reveal the anti-fibrotic mechanism of Sim in silica-induced rats, we added AICAR (an AMPK agonist) group and detected the expressions of phosphorylated AMPK (p-AMPK) and AMPK in lung tissues. As expected, treatment with Sim and AICAR both reversed the downregulated p-AMPK expression induced by silica in rats, indicating that Sim attenuated lung fibrosis in silica-induced rats possibly through the activation of AMPK (*p* < 0.05 or *p* < 0.01; Fig. 6A, B and Fig. S2.). To confirm the association between AMPK and NOX, we observed the activities of NOX in the Sim and AICAR groups and found that AMPK activation could down-regulate the activities of NOX and ROS in rats induced by silica (*p* < 0.05 or *p* < 0.01; Fig. 5A and B).

Activation of NOX depends on phosphorylation and stability of the subunits, thus we measured the expression of NOX subunits. It could be observed that treatment with AICAR and Sim (5, 10 mg/kg) could remarkably down-regulate the expressions of NOX4, p22phox, p40phox and p-p47phox in lung tissues of silica-induced rats (*p* < 0.05 or *p* < 0.01; Fig. 6C-H and Fig. S3). These results indicated that Sim may exert therapeutic effects by activating AMPK and downregulating NOX-derived ROS production.

**Fig. 7:**
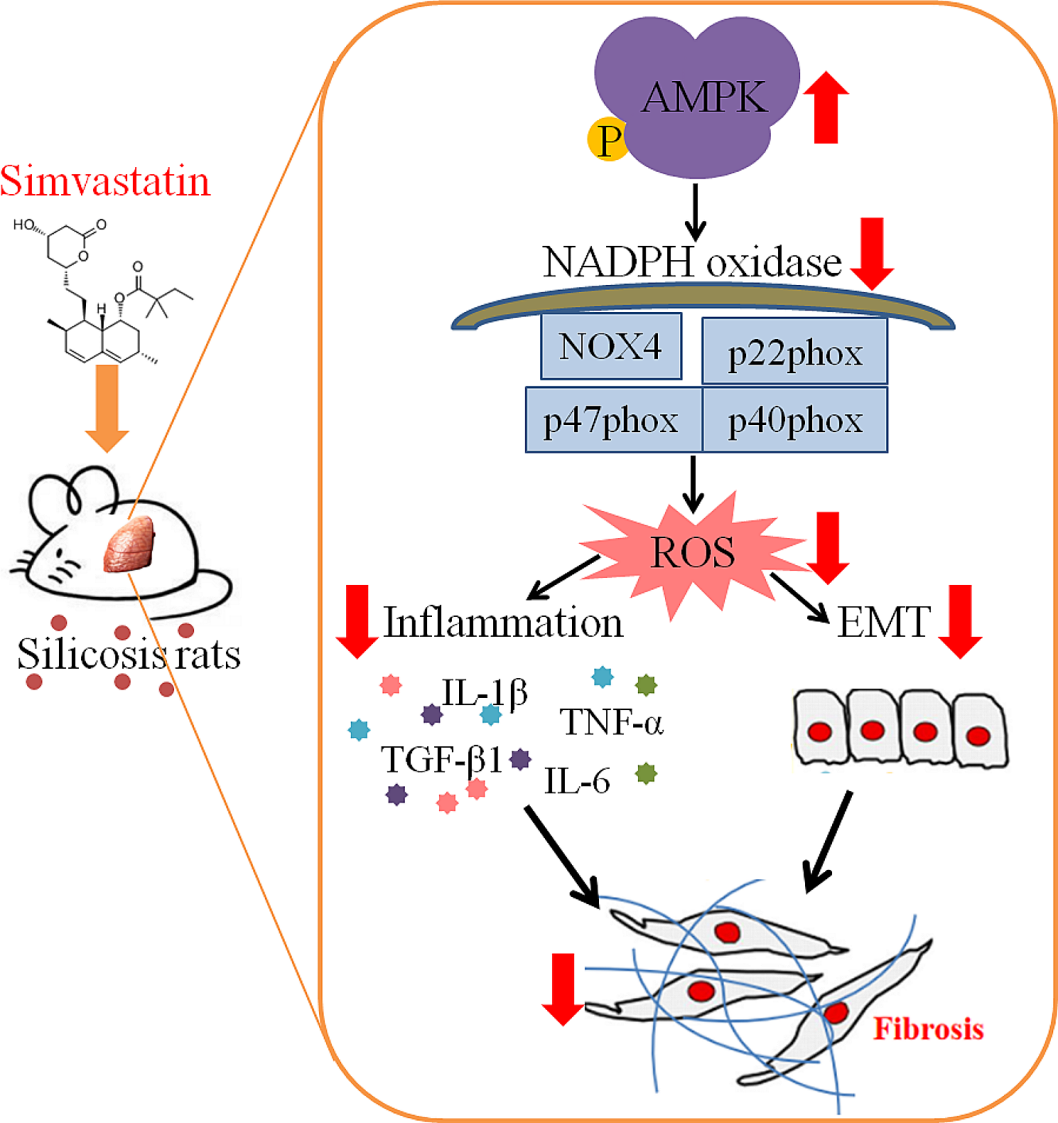
The signaling pathways of this experiment. Sim exerted protective effects against silica-induced pulmonary fibrosis in rats via the upregulation of p-AMPK expression and downregulation of NOX axis

## Discussion

OS and EMT play crucial roles in the pathogenesis of silicosis. Excessive ROS leads to lipid peroxidation, alveolar epithelial cell damage, DNA damage and apoptosis, thus contributing to the development of silicosis. Due to the complexity of silicosis, there are few therapeutic strategies to date. Therefore, it is of great significance to study the pathogenesis of silicosis and explore effective drugs. Sim, an inhibitor of 3-hydroxy-3-methylglutaryl coenzyme A used to treat atherosclerotic diseases, has protective effects on bleomycin-induced pulmonary fibrosis in rats by reducing the levels of IL-13 and TGF-β1 [43]. Moreover, Sim has anti-fibrotic properties by modulating the expression of Yes-associated proteins mediating the activation of mouse fibroblasts [44]. However, few studies are designed to explore the effects and potential mechanisms of Sim in silica-induced rats. In our study, the results showed that silica exposure for 28 days could cause lung inflammation, OS and fibrosis, accompanying by the increase of NOX and ROS activities, and decrease of p-AMPK expression in lung tissues. In parallel, Sim treatment could significantly attenuate these silica-induced changes in rats. All these findings suggest that Sim treatment alleviates silica-induced pulmonary fibrosis through activation of AMPK and down-regulation of NOX expression, providing an innovative option for the treatment of silicosis.

Sim is commonly used as a lipid-lowering drug, with the dosage of 5 mg/kg and 10 mg/kg as described previously [38]. In the present study, treatment with Sim (5 or 10 mg/kg) ameliorated silica-induced pulmonary inflammation and fibrosis in rats, without changes in serum levels of TCHO, TG, HDL-C and LDL-C. Silica exposure caused structural damage to the lungs and significantly increased lung coefficients. At the same time, we observed that the administration of Sim, AICAR and apocynin significantly alleviated pathological damage of lung tissues and reduced lung coefficients in rats.

It has been reported that silica exposure initially induces inflammation in lung tissues, and promotes the release of inflammatory factors such as IL-1β, IL-6 and TNF-α to participate in inflammatory and immune responses, consequently leading to the development of silicosis fibrosis [45]. Our data showed that the IL-1β, IL-6 and TNF-α levels in lung tissues were significantly increased after 28 days of silica exposure, while remarkably decreased after treatment with Sim. H&E staining further confirmed that silica exposure for 28 days induced obvious lung pathological damage, including thickened alveolar walls, enlarged alveolar intervals, and apparent inflammatory cell infiltration. According to the quantified score of alveolar inflammation, we found that the scores of alveolitis significantly increased after silica exposure. However, Sim treatment could significantly alleviate these abnormal pathological damages and decrease the score of alveolar inflammation in silica-induced rats. These results unveil that silica exposure can cause lung injury and inflammation, which can be significantly alleviated by Sim treatment.

Progressive pulmonary fibrosis is the most important characteristic in the advanced stage of silicosis [26]. Collagen, the major component of ECM, reflects the degree of fibrosis. As a unique amino acid in collagens, HYP can reflect the degree of pulmonary fibrosis. In our study, the expression of HYP was significantly increased after 28 days of silica exposure, but notably reduced after treatment with Sim. Additionally, we also demonstrated that Sim could significantly decrease the area of collagen deposition and fibrosis scores in lung tissues of silica rats. Hence, Sim treatment is conductive to inhibiting the lung fibrosis induced by silica.

EMT is a process of pulmonary fibrosis during which alveolar epithelial cells lose their identity and transform into mesenchymal epithelial cells, accompanying by downregulated expression of E-cad and upregulated expressions of α-SMA and Vimentin. Previous studies have indicated that the EMT exerts a critical effect on promoting pulmonary fibrosis [46–47]. Moreover, TGF-β1 plays an important role in the induction of EMT [48]. To explore the anti-fibrotic effect of Sim in silica-induced rats, TGF-β1 and EMT-associated protein levels were detected. We found that silica exposure significantly increased the expressions of TGF-β1, α-SMA and Vimentin, but decreased the expression of E-cad in lung tissues, implying that the EMT process occurred in lung tissues after silica exposure. However, after treatment with Sim, the expression of E-cad was remarkably increased while the expressions of α-SMA and Vimentin were significantly decreased, suggesting that Sim may alleviate pulmonary fibrosis by inhibiting the EMT process.

The degree of OS depends on the imbalance between production and deletion of ROS. Excessive ROS causes lipid peroxidation to damage the lung epithelium and promote fibrosis. ROS also acts as a signaling molecule to activate inflammatory pathways and promote the EMT process. NOX family is the only enzyme directly producing ROS in various organs, among which NOX2 and NOX4 are mainly expressed in the lungs [6, 49]. NOX4 plays a role in some lung diseases, such as acute respiratory distress syndrome, chronic obstructive pulmonary disease and pulmonary fibrosis [49]. The study reported LPS-induced OS and inflammation were alleviated by reducing NOX2 activity in lung epithelial A549 cells [6]. In our study, the expression of NOX4 was significantly increased after 28 days of silica exposure, while the NOX2 expression had no changes in silica-induced rat, which was inconsistent with the results of upregulated NOX2 mRNA expression in silica-induced pulmonary fibrosis [50]. This may be attributed to inconsistent expression between the gene expression and protein levels. The activation of NOX2 mainly depends on the stability and phosphorylation of subunits. By assessing the expressions of p22phox, p40phox and p-p47phox subunits in silica-induced pulmonary fibrosis, we proved that activation of the NOX2 may be attributed to the phosphorylation of p47phox, supporting the importance of NOX-derived ROS in the pathogenesis of silicosis.

Apocynin, a natural inhibitor of NADPH oxidase, plays its role mainly by reducing NOX-derived ROS. In our study, both Sim and apocynin could alleviate the levels of OS by downregulating the level of MDA and up-regulating the activities of SOD and GSH-Px through inhibition of NOX and ROS expressions, suggesting that the anti-OS effects of Sim on the lung tissue in silica-induced rats may be related to the inhibition of NOX and its derived ROS. ROS, a signaling molecule, plays a vital role in the development of inflammatory diseases, and its inhibition can reduce the activation of NLRP3 inflammasome pathway to protect LPS-induced renal injury [51–52]. After treatment with apocynin and Sim, inflammatory cell infiltration and expressions of pro-inflammatory factors (IL-1β, IL-6, and TNF-α) were all decreased in lung tissues after silica exposure. It is believed that the palliative effects of Sim on inflammation in silica-induced rats may be partly related to the inhibition of NOX-derived ROS. In addition, OS also can trigger DNA damage and cell death. Lung epithelial cell death/apoptosis is one of the critical pathophysiologic events, limiting normal lung repair and facilitating pulmonary fibrosis, which occurs in the patients with idiopathic pulmonary fibrosis (IPF) and animal models of pulmonary fibrosis [53]. NOX4 is upregulated and strongly expressed in alveolar type 2 cells of mice treated with bleomycin and patients with IPF, and hydrogen peroxide produced by NOX4 promotes AEC death [54–56]. ROS-induced DNA damage can trigger apoptosis and contribute to the fibrogenesis of OS. NOX4-derived ROS arbitrates TGF-β1-induced metabolic reprogramming during EMT via PI3K/AKT/HIF-1α pathway in glioblastoma [57]. TGF-β, the most potent profibrogenic cytokine, stimulates ROS production, leading to OS. Oppositely, OS can activate latent TGF-β, which sets up a vicious profibrogenic circle to aggravate the development of pulmonary fibrosis [58]. Additionally, TGF-β1 can also induce the EMT process in alveolar epithelial cells [59]. In our study, apocynin and Sim significantly increased the expression of E-cad, while decreased the expressions of TGF-β1, α-SMA and Vimentin in lung tissues, suggesting that Sim could alleviate the silica-induced lung fibrosis by inhibiting NOX-derived ROS production and reducing TGF-β1-induced EMT process. Whether Sim treatment could inhibit lung epithelial cell apoptosis needs to be further investigated in future studies.

AMPK is considered to be a cellular energy sensor, consisting of α, β, and γ subunits and expressed in the heart, lungs and kidneys [16–18]. Its activation mainly depends on the phosphorylation of AMPKα subunit. Studies has shown that AMPK activation can inhibit the synthesis of TNF-α, IL-1β and IL-6 in macrophages [60–61], and ameliorate OS-induced senescence [62]. Moreover, a close association between AMPK and cardiac or pulmonary fibrosis has been indicated. In the AMPKα1 knockout mouse, myocardial fibroblast proliferation and migration were enhanced, while myocardial differentiation was inhibited, suggesting lack of AMPK would promote the cardiac fibrosis [19]. In the animal model, low AMPK activity could also be found to promote pulmonary fibrosis [25–26], and the metformin as an AMPK agonist could alleviate silica-induced lung inflammation and fibrosis by regulating autophagy through AMPK activation and mTOR inhibition [26]. A previous study has shown that Sim can protect diabetes mellitus-induced erectile dysfunction by promoting AMPK pathway [63], and can decrease the contractility of isolated mesenteric resistant arteries in rats through activation of AMPK [33]. Therefore, we hypothesized that Sim treatment might alleviate silica-induced lung injury and fibrosis in rats by enhancing phosphorylated AMPK expression. AICAR, a classical agonist of AMPK, has been presented to attenuate liver injury and fibrosis in bile duct ligation rats by triggering AMPK [64]. Consistent with previous results, p-AMPK expression was significantly decreased after silica exposure for 28 days in our study [26], and treatment with AICAR and Sim notably attenuated silica-induced lung damage and fibrosis via activation of AMPK, suggesting that Sim may have similar effects to AICAR.

Notably, AMPK plays a pivotal role in renal vasodilatation through AMPK-NOX signaling, and the combination of linagliptin and metformin could improve neointimal hyperplasia in diabetic rats by regulating AMPK/NOX4 signaling pathway [27, 65]. Additionally, activation of AMPK could limit mitochondrial ROS production and trigger a PGC-1α-dependent antioxidant response [66]. In this study, we found that AICAR and apocynin both remarkably alleviated lung inflammation, OS and fibrosis in silica-induced rats, indicating that Sim may have similar capabilities to AICAR and apocynin by activating AMPK or preventing activities of NOX and ROS. Thus, it is reasonable to believe that Sim may alleviate silica-induced pulmonary fibrosis by activating AMPK and inhibiting NOX and its derived ROS production.

## Conclusions

Our study demonstrated that Sim has anti-silicotic effects in rats. The effects of Sim may be associated with its abilities to alleviate the degree of alveolitis and pulmonary fibrosis, inhibit EMT and OS. Importantly, Sim can attenuate silica-induced pulmonary inflammation and fibrosis by activating AMPK and inhibiting NOX signaling, and the mechanism was shown in Fig. 7. These findings may provide an additional therapeutic option for patients with silicosis, especially for those co-existing with cardiovascular diseases. Whether Sim can be a treatment option in patients with pulmonary fibrosis needs to be investigated in further studies.

### Electronic supplementary material

Below is the link to the electronic supplementary material.

Supplementary Material 1

Supplementary Material 2

Supplementary Material 3

Supplementary Material 4

## Data Availability

The datasets used and/or analyzed during the current study are available from the corresponding author on reasonable request.

## Abbreviations

Sim: Simvastatin
NADPH: Nicotinamide adenine dinucleotide phosphate
NOXs: NADPH oxidase
ROS: reactive oxygen species
OS: oxidative stress
TGF-β1: transforming growth factor-β1
EMT: epithelial-mesenchymal transition
ECM: extracellular matrix
AMPK: Adenosine 5’-monophosphate (AMP)-activated protein kinase
SPF: specific pathogen-free
TCHO: total cholesterol
TG: triglycerides
LDL-C: low density liptein cholesterol
HDL-C: high density liptein cholesterol
H&E: hematoxylin and eosin
SOD: Superoxide dismutase
GSH-Px: Glutathione peroxidase
MDA: Malondialdehyde
