# Supplementary figures and images for: Simvastatin attenuates silica-induced pulmonary inflammation and fibrosis in rats via the AMPK-NOX pathway
Source: BMC Pulm Med. 2024 May 8;24:224. doi: 10.1186/s12890-024-03014-9 (PMC11080310; doi:10.1186/s12890-024-03014-9)

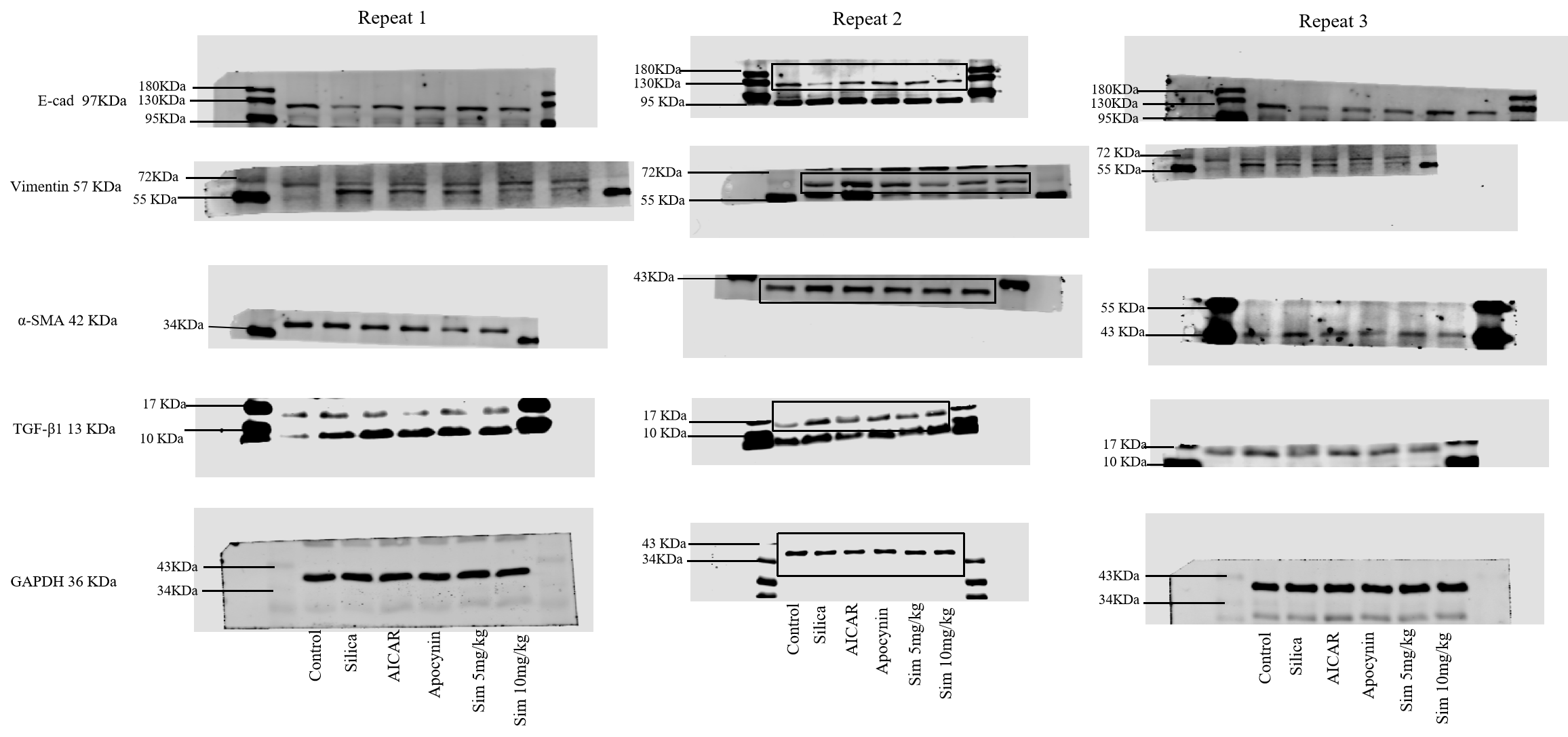

Supplement: Supplementary file 1 — Supplementary Material 1 [file 12890_2024_3014_MOESM1_ESM.tif]

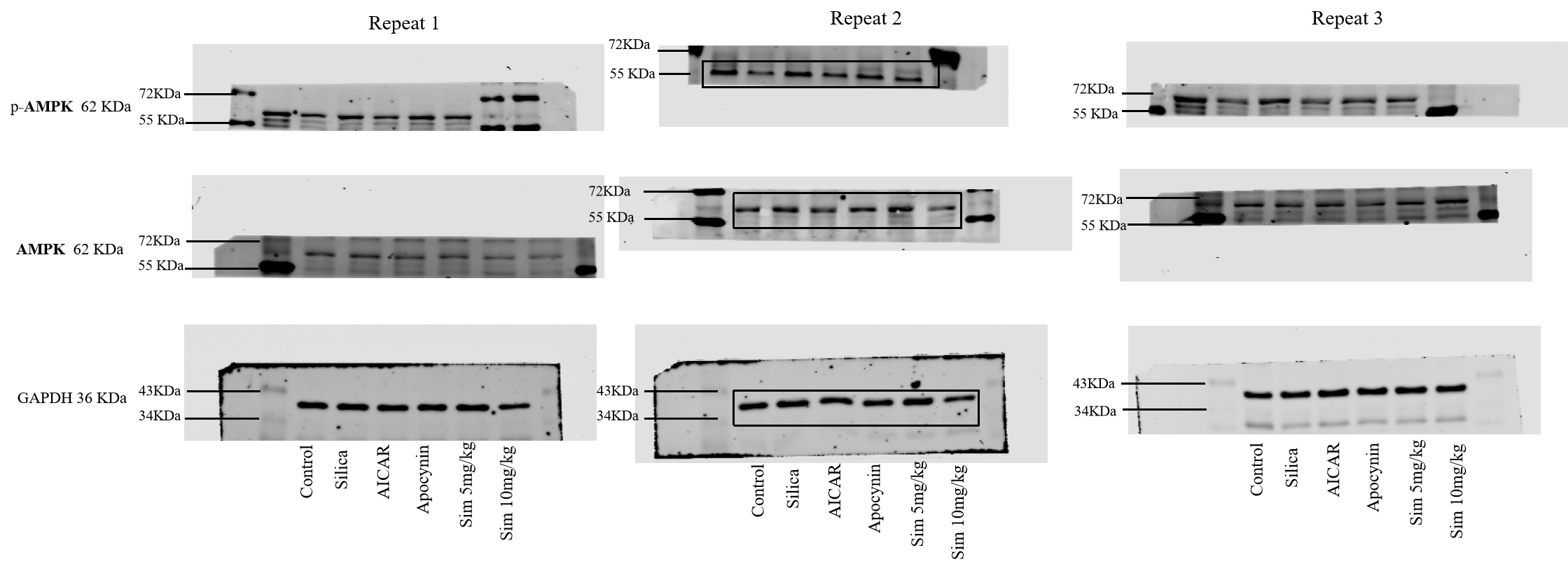

Supplement: Supplementary file 2 — Supplementary Material 2 [file 12890_2024_3014_MOESM2_ESM.tif]

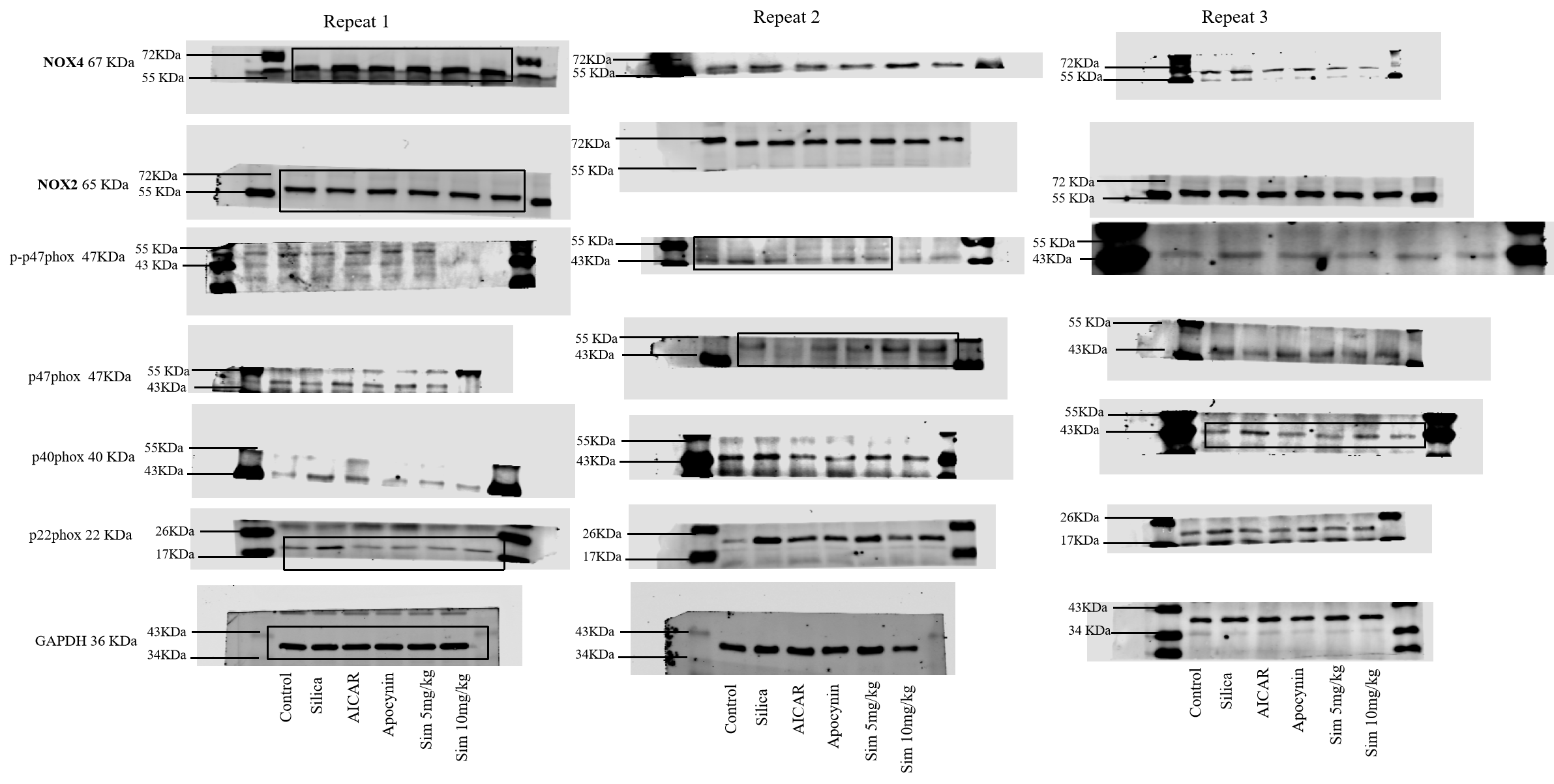

Supplement: Supplementary file 3 — Supplementary Material 3 [file 12890_2024_3014_MOESM3_ESM.tif]
