# Supplementary material for: Simvastatin attenuates silica-induced pulmonary inflammation and fibrosis in rats via the AMPK-NOX pathway
Source: BMC Pulm Med. 2024 May 8;24:224. doi: 10.1186/s12890-024-03014-9 (PMC11080310; doi:10.1186/s12890-024-03014-9)
Supplement: Supplementary file 4 — Supplementary Material 4 [file 12890_2024_3014_MOESM4_ESM.doc]

**Supplementary figure legends**

**Figure S1.** Western blot images of Fig. 4A. The WB legends of the rats lung tissue. The blots were cut prior to hybridisation with antibodies during blotting. The images show all blots and replicates of E-cad, Vimentin, α-SMA, TGF-β1, and GAPDH. The inner black rectangle are the representative blots

**Figure S2.** Western blot images of Fig. 6A. The WB legends of the rats lung tissue. The blots were cut prior to hybridisation with antibodies during blotting. The images show all blots and replicates of p-AMPK, AMPK , and GAPDH. The inner black rectangle are the representative blots.

**Figure S3.** Western blot images of Fig. 6C. The WB legends of the rats lung tissue. The blots were cut prior to hybridisation with antibodies during blotting. The images show all blots and replicates of NOX4, NOX2, p-p47phox, p47phox, p40phox, p22phox, and GAPDH. The inner black rectangle are the representative blots.
